# Subgradient-projection-based stable phase-retrieval algorithm for X-ray ptychography

**DOI:** 10.1107/S1600576724004709

**Published:** 2024-07-04

**Authors:** Natsuki Akaishi, Koki Yamada, Kohei Yatabe, Yuki Takayama

**Affiliations:** a Department of Electrical Engineering and Computer Science Tokyo University of Agriculture and Technology 2-24-16 Naka-cho, Koganei Tokyo Japan; b International Center for Synchrotron Radiation Innovation Smart, Tohoku University, 2-2-1 Katahira, Aoba-ku, Sendai, Miyagi, Japan; c Graduate School of Agricultural Science, Tohoku University, 468-1 Aoba-ku, Sendai, Japan; d Research Center for Green X-Tech, Green Goals Initiative, Tohoku University, 6-6 Aoba-ku, Sendai, Japan; e RIKEN SPring-8 Center, 1-1-1 Kohto, Sayo, Sayo-gun, Hyogo, Japan; Ecole National Supérieure des Mines, Saint-Etienne, France

**Keywords:** hard X-ray ptychography, phase retrieval, subgradient projection

## Abstract

X-ray ptychography is a lensless imaging technique that visualizes the nano­structure of a thick specimen which cannot be observed with an electron microscope. It reconstructs a complex-valued refractive index of the specimen from observed diffraction patterns. This reconstruction problem is called phase retrieval (PR). For further improvement in the imaging capability, including expansion of the depth of field, various PR algorithms have been proposed. Since a high-quality PR method is built upon a base PR algorithm such as ePIE, developing a well performing base PR algorithm is important. This paper proposes an improved iterative algorithm named CRISP. It exploits subgradient projection which allows adaptive step size and can be expected to avoid yielding a poor image. The proposed algorithm was compared with ePIE, which is a simple and fast-convergence algorithm, and its modified algorithm, rPIE. The experiments confirmed that the proposed method improved the reconstruction performance for both simulation and real data.

## Abstract 2

This paper proposes a phase-retrieval algorithm for X-ray ptychography with almost the same computational efficiency as the conventional methods. It exploits an optimization technique, subgradient projection, which has an interesting property that means it can be expected to avoid yielding poor images.

## 1. Introduction

Ptychography is a lensless imaging technique for microscopic observation, based on the diffraction of a coherent wave, including X-rays (Maiden & Rodenburg, 2009 ▸; Harada *et al.*, 2013 ▸; Valzania *et al.*, 2018 ▸). With the increasing demand for nondestructive high-spatial-resolution imaging of various specimens, hard X-ray ptychography has gained much attention. The short wavelength and high penetrability of hard X-rays enable nondestructive observation of internal structures of thick specimens at high spatial resolution, even though they are too thick for electron microscopy. Hard X-ray ptychography has been applied to specimens in various fields including biology (Polo *et al.*, 2020 ▸; Suzuki *et al.*, 2016 ▸; Shahmoradian *et al.*, 2017 ▸; Jones *et al.*, 2014 ▸), chemistry (Pattammattel *et al.*, 2020 ▸; Shi *et al.*, 2019 ▸; Hirose *et al.*, 2019 ▸, 2020 ▸) and materials science (Cuesta *et al.*, 2019 ▸; Grote *et al.*, 2022 ▸; Uematsu *et al.*, 2021 ▸; Gao *et al.*, 2020 ▸).

**Figure 1:**
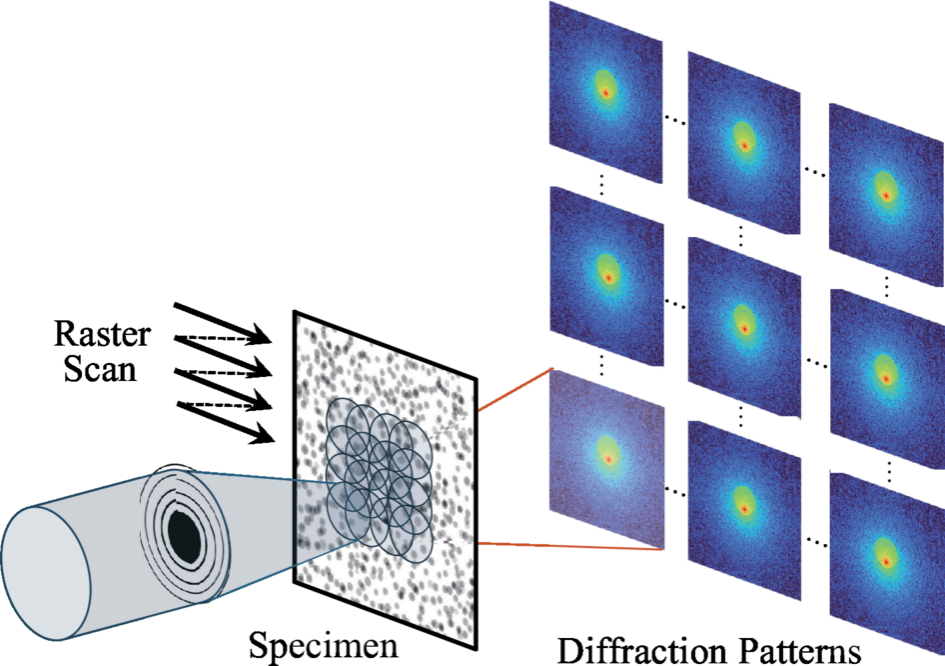
Schematic illustration of a ptychographic measurement.

Fig. 1 ▸ schematically illustrates the ptychographic measurement in transmission geometry. In the measurement, Fraunhofer diffraction patterns from the specimen (termed ‘object’) are collected with a localized X-ray beam (the ‘probe’). This measurement is repeated by shifting the illumination area on the object with some overlap. The diffraction data set is then subjected to a computational image reconstruction process utilizing an iterative optimization algorithm, yielding the spatial distribution of the complex-valued refractive index (*i.e.* phase and absorption contrast image) of the object and also the complex wavefield of the probe. This image reconstruction process is often called phase retrieval (PR) because the process recovers phase information of the diffraction wavefield lost in the diffraction data.

Various algorithms have been proposed to solve the PR problem in ptychography: the conjugate gradient method (Guizar-Sicairos & Fienup, 2008 ▸), the difference map (Elser, 2003 ▸; Thibault *et al.*, 2009 ▸), relaxed averaged alternating reflections (Luke, 2004 ▸; Marchesini *et al.*, 2016 ▸), the maximum likelihood method (Thibault & Guizar-Sicairos, 2012 ▸), the proximal splitting algorithm (Hesse *et al.*, 2015 ▸; Qian *et al.*, 2014 ▸) and the extended ptychographical iterative engine (ePIE) (Rodenburg & Faulkner, 2004 ▸; Maiden & Rodenburg, 2009 ▸). In the iterative update of the object and probe functions, most algorithms use the entire set of diffraction patterns simultaneously, while ePIE sequentially reflects the diffraction patterns point by point. The former batch updating approach is advantageous for parallel computing, but it requires more memory compared with the latter sequential updating approach and is prone to a poor local solution (Pham *et al.*, 2019 ▸). It is also empirically known that the batch updating approach can actually require more iterations for convergence (Yatabe & Takayama, 2022 ▸). On the other hand, the ePIE algorithm is simple and computationally efficient and thus widely used.

Because of the favorable properties of the ePIE algorithm, it is also used for advanced PR. Advanced PR tackles, for example, a practical restriction that the higher the spatial resolution, the thinner the sample must be. This trade-off relationship between sample thickness (depth of field) and spatial resolution is known as the diffraction limit in the theory of general transmission microscopy (Tsai *et al.*, 2016 ▸; Born & Wolf, 1980 ▸). To overcome this trade-off relationship, a multi-slice PR approach has been proposed as an extension of ePIE (Tsai *et al.*, 2016 ▸; Maiden *et al.*, 2012 ▸). Thanks to the strong real-space constraint in ptychography, multi-slice PR achieved extension of the depth of field. However, the increase in information to be retrieved in the multi-slice approach makes the PR problem more difficult, and reconstructed-object slices often suffer from cross-talk artifacts caused by poor depth resolution (Du *et al.*, 2021 ▸). To reduce the artifacts, it is important to develop a base PR algorithm that performs better than ePIE.

To improve the convergence performance of ePIE, some variants have been proposed (Maiden & Rodenburg, 2009 ▸; Maiden *et al.*, 2017 ▸; Pham *et al.*, 2019 ▸). The regularized PIE (rPIE) (Maiden *et al.*, 2017 ▸) involves a modification of the updating formula of ePIE with a regularization weighting that uses the square of the absolute value of the object and probe. The momentum PIE (mPIE) improves the convergence speed with a momentum motivated by a stochastic or incremental gradient approach (Maiden *et al.*, 2017 ▸). Although rPIE and mPIE may work better with well tuned hyperparameters, it is difficult to tune them because their heuristic modifications involve an increase in the number of hyperparameters.

In this paper, we propose a stable PIE-like algorithm named CRISP to realize a reliable method that can automatically tune its hyperparameter. CRISP exploits an optimization technique that is called subgradient projection. Thanks to the favorable property of subgradient projection, CRISP can avoid getting stuck in poor local solutions. Moreover, an auxiliary method included in the proposed method can automatically tune the parameter for subgradient projection. With the combination of these techniques, CRISP achieves high performance despite its simplicity. Our experiments showed that CRISP improved reliability especially at high spatial frequencies and reduced PR artifacts while achieving convergence speeds comparable to those of ePIE and rPIE.

## 2. Preliminaries

### 2.1. Notation

The two-dimensional Fourier transform operator and the two-dimensional inverse Fourier transform operator are denoted by 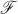 and 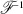, respectively. Matrices are indicated by bold capital letters, *i.e.***A**, and the elements of the *i*th row and *j*th column are denoted by *A*[*i*, *j*]. Vectors are indicated by bold lower-case letters, *i.e.***v**, and the *i*th element is denoted by *v*[*i*]. Element-wise multiplication is denoted by  ⊙ , the element-wise absolute value is denoted by | · | and the largest absolute value among all elements of input is denoted by | · |_max_. ∥**A**∥_F_ is the Frobenius norm, which is defined as 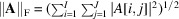. sign(·) denotes the signum function that is generalized for complex numbers as sign(*z*) = *z*/|*z*|. The complex conjugate of **A** is denoted by 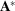. For any differential function *g*, ∇*g* denotes the gradient of *g*.

### 2.2. Ptychographical iterative engine framework

**Figure 2:**
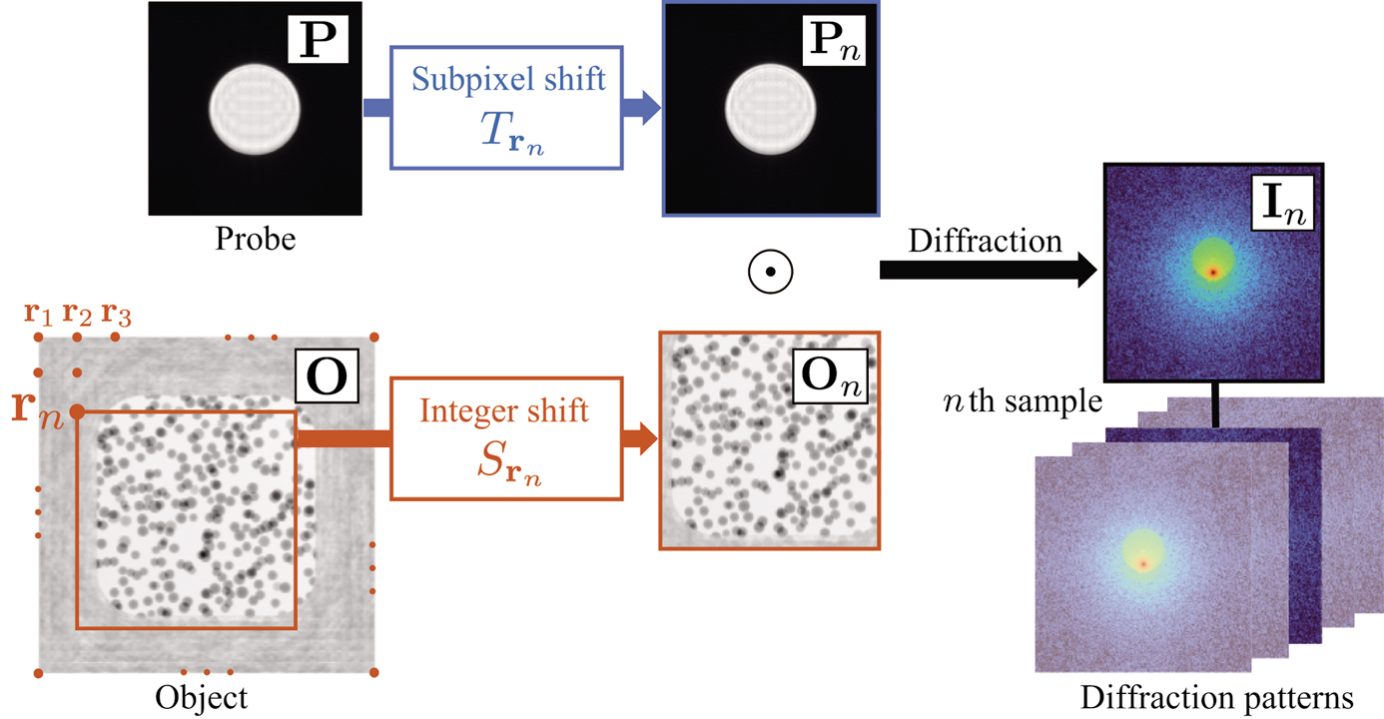
Schematic illustration of the observation model.

We first explain the ptychographic measurement model shown in Fig. 2 ▸. In ptychographic measurement, a set of diffraction patterns is observed by shifting the measurement position. The *n*th observed diffraction intensity pattern **I**_*n*_ corresponds to the squared magnitude of the two-dimensional Fourier transform of the exit wavefield. The wavefield is modeled by multiplication of the illumination probe function **P** and the object transmission function **O**. Thus, the *n*th observed diffraction intensity pattern **I**_*n*_ can be written as 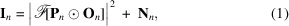
where *n* = 1,…, *N* is a sample index, *N* is the number of samples, 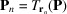, 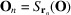, and **N**_*n*_ is an observation’s noise. The functions 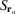 and 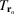 model the position shift with the coordinate vector of the *n*th scan position **r**_*n*_. The position shift is decomposed into integer and subpixel shifting. The function 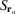 performs an integer shift, and 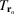 performs a subpixel shift.

Ptychography aims to estimate the object transmission and illumination probe functions from the observed diffraction patterns. In the PIE-based algorithms, the PR problem can be formulated as the following optimization problem that minimizes the squared error in the exit wavefield in the real space: 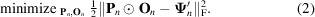

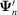
 is the revised exit wavefield constrained to the reciprocal space (Maiden *et al.*, 2017 ▸): 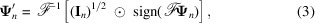
where 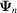 is the exit wavefield of the *n*th scan position, *i.e.*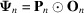. This manipulation replaces the propagated modulus with the square root of the observed diffraction patterns and propagates back to the real space. It is difficult to find a reasonable solution to the problem in equation (2) because it involves the product of unknown variables 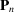 and **O**_*n*_ and has local minima. To make the problem simpler, the PIE-based algorithms deal with the following two sub­problems that optimize each variable **O**_*n*_ and 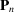 while fixing the other ones: 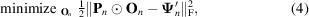

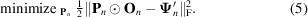
ePIE (Maiden & Rodenburg, 2009 ▸) updates the object (o) and probe (p) as follows: 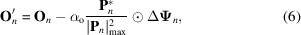

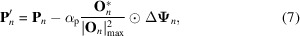
where α_o_, α_p_ ∈ (0, 1] are step size parameters, and 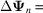

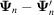
. The update formulas can be obtained by applying the gradient descent method to each subproblem in equations (4) and (5) (details will be given in the following section).

rPIE (Maiden *et al.*, 2017 ▸) considers the slightly different subproblems in equations (4) and (5) and includes a regularization term with the subproblems such as 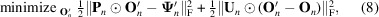

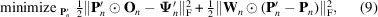
where **U**_*n*_ and **W**_*n*_ are weight matrices with nonnegative elements. The 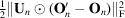 and 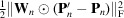 terms penalize significant changes to objects and probes between updates. By setting the weights as 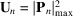 and 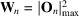 and solving the subproblems in equations (8) and (9), the updating formulas of rPIE can be derived as follows: 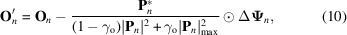

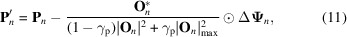
where γ_o_, γ_p_ > 0 are balancing parameters, and division is computed element-wise.

The entire procedure of ePIE is summarized in the following pseudocode, where *k* = 1,…, *K* is the iteration index, *K* is the number of iterations, **l** is a vector of the randomized sample indices, and 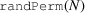 is a function that randomly permutes an integer vector up to *N*. The function 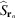 assigns the object sampled by the function 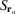 to the scan position **r**_*n*_, and the function 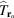 restores the probe shifted by the function 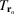. The updating formulas, equations (6) and (7), are on lines 11 and 12. When lines 11 and 12 are changed to equations (10) and (11), the procedure becomes the rPIE algorithm.
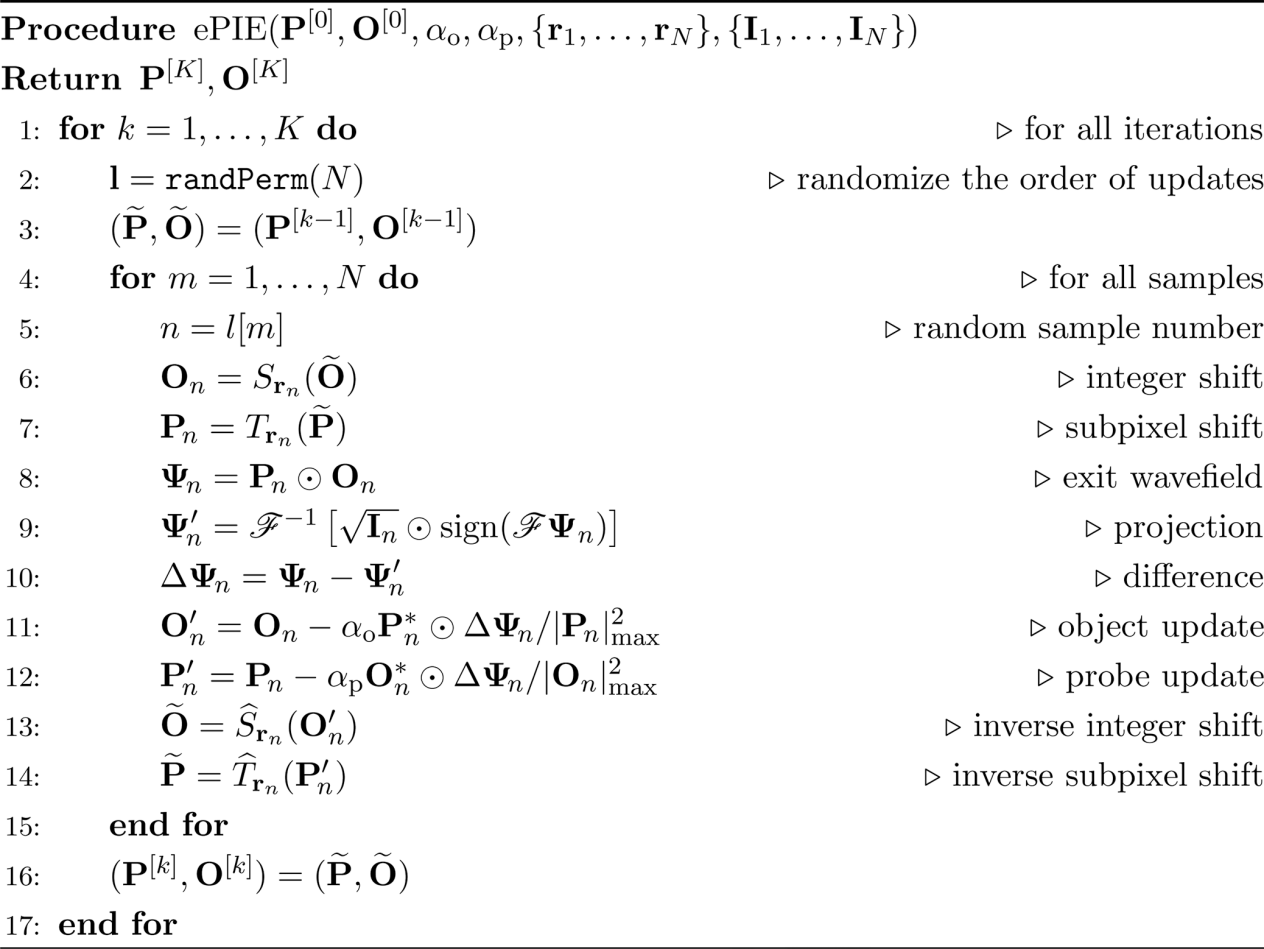


### 2.3. ePIE as a gradient descent method

As mentioned in the previous section, the updating formulas of ePIE are based on the gradient descent method. It is helpful for characterizing the proposed method to explain the derivation of ePIE’s updating formulas. In particular, the property of its step size will give a suggestion for the proposed method.

Let **x** be an optimization variable and *g* a differentiable cost function to be minimized. The gradient descent method finds a local minimum of *g* by iterating the following update: 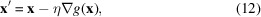
where **x** and **x**′ are, respectively, variables before and after the update, and η is a step size parameter. By applying equation (12) to equation (4), the following updating formula of **O**_*n*_ is obtained: 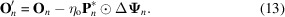
In the same manner, from equation (5) and (12) the updating formula of 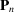 is obtained: 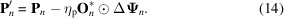
If we set the step size parameters 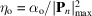 for equation (13) and 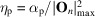 for equation (14), these updating formulas become equal to equations (6) and (7).

The step size setting of ePIE is derived from the property that *g*(**x**′) < *g*(**x**) if η ∈ (0, 1/*L*], where *L* is the Lipshitz constant of ∇*g*(**x**) (Bauschke & Combettes, 2017 ▸). This property guarantees the cost always decreases through updates. The Lipshitz constants of the gradient of the cost functions in equations (4) and (5) are given by 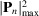 and 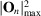, respectively (Qian *et al.*, 2014 ▸). Thus, the updating formulas in equations (13) and (14) always decrease the cost under the conditions 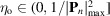 and 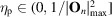. In the update of ePIE, setting α_o_, α_p_ ∈ (0, 1] satisfies the condition. In practice, it has been shown that ePIE stably converges when α_o_, α_p_ is chosen from the range (0, 1] (Maiden *et al.*, 2017 ▸).

## 3. Proposed method

We propose a PR algorithm, CRISP (clipped reliable iterative subgradient projection). We exploit a subgradient projection that is considered to have a favorable property (Section 3.3). The proposed method consists of a main updating formula and two auxiliary methods that improve the stability (Section 3.4) and simplicity (Section 3.5) of the proposed method. The explanations of these auxiliary methods follow an intuitive explanation of the subgradient-projection-based algorithm.

### 3.1. Subgradient projection

We first introduce subgradient projection, which plays a central role in the proposed method. Subgradient projection is known in the field of optimization and is usually used for optimization with non-differentiable cost functions whose subgradient can be calculated. We introduce the simplified version of subgradient projection that is for differentiable functions because we only consider a differentiable function in this paper.

Let *g* be a differentiable function. The subgradient projection for a differentiable function is given by 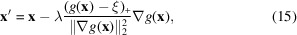
where (·)_+_ = max{0, ·}, λ ∈ (0, 2) is a step size parameter and ξ is a real-valued hyperparameter (Bauschke & Combettes, 2017 ▸).

The iterative subgradient projection algorithm (Bauschke & Combettes, 2017 ▸), which uses subgradient projection iteratively, can be regarded as a type of gradient descent method. Compared with the updating formula in equation (12), the step size η is replaced by a scalar-valued function 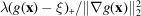. From now on, we treat this scalar variable as the step size of the updating formula of iterative subgradient projection.

### 3.2. The basic form of CRISP

We next derive a basic updating formula based on subgradient projection. As introduced in Section 2.2, the cost function of the PR problem in ptychography is 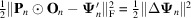. Applying the cost function for each variable **O**_*n*_ and 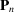 to the formula of the subgradient projection in equation (15), the following formulas can be obtained: 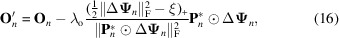

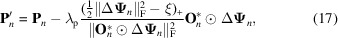
where λ_o_, λ_p_ are step size parameters with the same role as the step size parameters of ePIE (α_o_, α_p_), and ξ is a hyperparameter. We use the same ξ for all *n*.

An important property of the proposed updating formula is that the step size changes adaptively. The main part of the step size 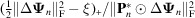 changes depending on the cost 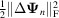.

This property can bring a better solution (details are given in the following section).

### 3.3. Intuitive explanation of CRISP

**Figure 3:**
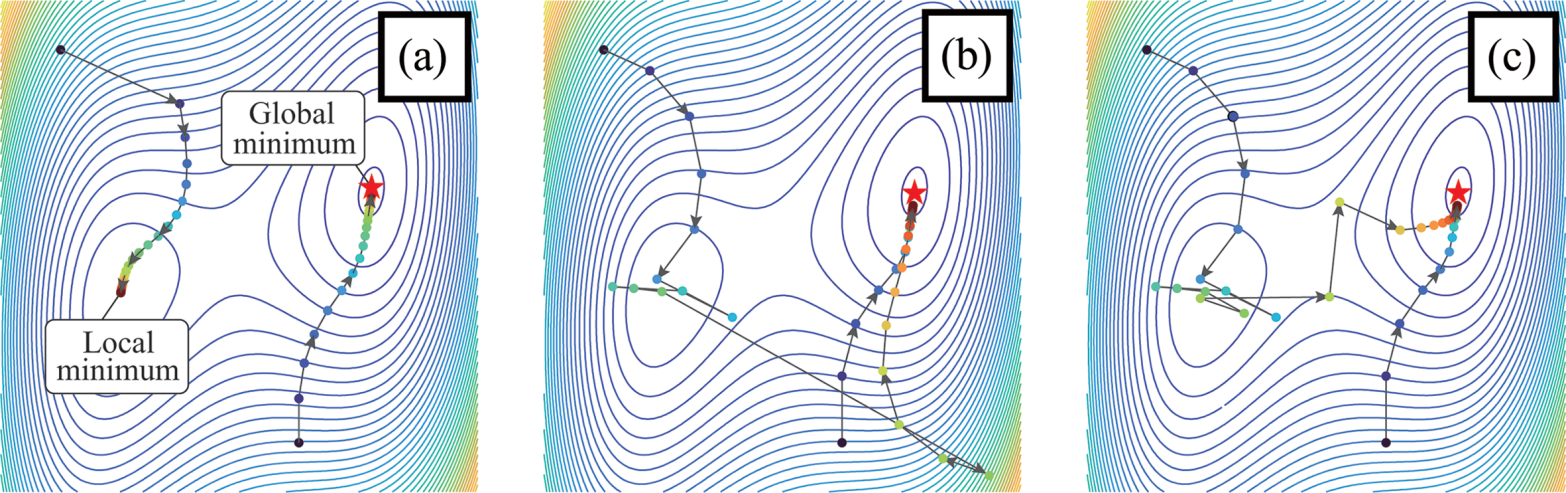
Contour plot of a cost function and trajectories of iterative updates. (*a*) An example using the gradient descent method. Examples using the iterative subgradient projection (*b*) without step size clipping and (*c*) with it. The points are colored according to the number of updates, and the arrows indicate the transition of each update. The global minimum is shown with a red star.

Let us clarify the properties of the updating formulas in equations (16) and (17). We first show that the iterative update using subgradient projection can bring about benefits that are likely to reach a better solution. In Fig. 3 ▸, an iterative update by the gradient descent method (corresponding to ePIE) and the subgradient projection (corresponding to CRISP) are demonstrated. As shown in Figs. 3 ▸(*a*) and 3 ▸(*b*), the gradient descent can get stuck in the local minimum, while the subgradient projection arrives at the global minimum even when it starts from a poor initial value. This is because the gradient descent method stops when ∇*g*(**x**) = 0 even if it is a local minimum whose cost is large. On the other hand, the subgradient projection keeps updating whenever *g*(**x**) > ξ. When the sequence of the subgradient projection approaches the local minimum, ∥∇*g*(**x**)∥ becomes close to 0, while *g*(**x**) is still larger than ξ. This makes the step size large because the step size includes the reciprocal of the gradient ∇*g*(**x**) as in equation (15). This property can avoid poor local minima.

**Figure 4:**
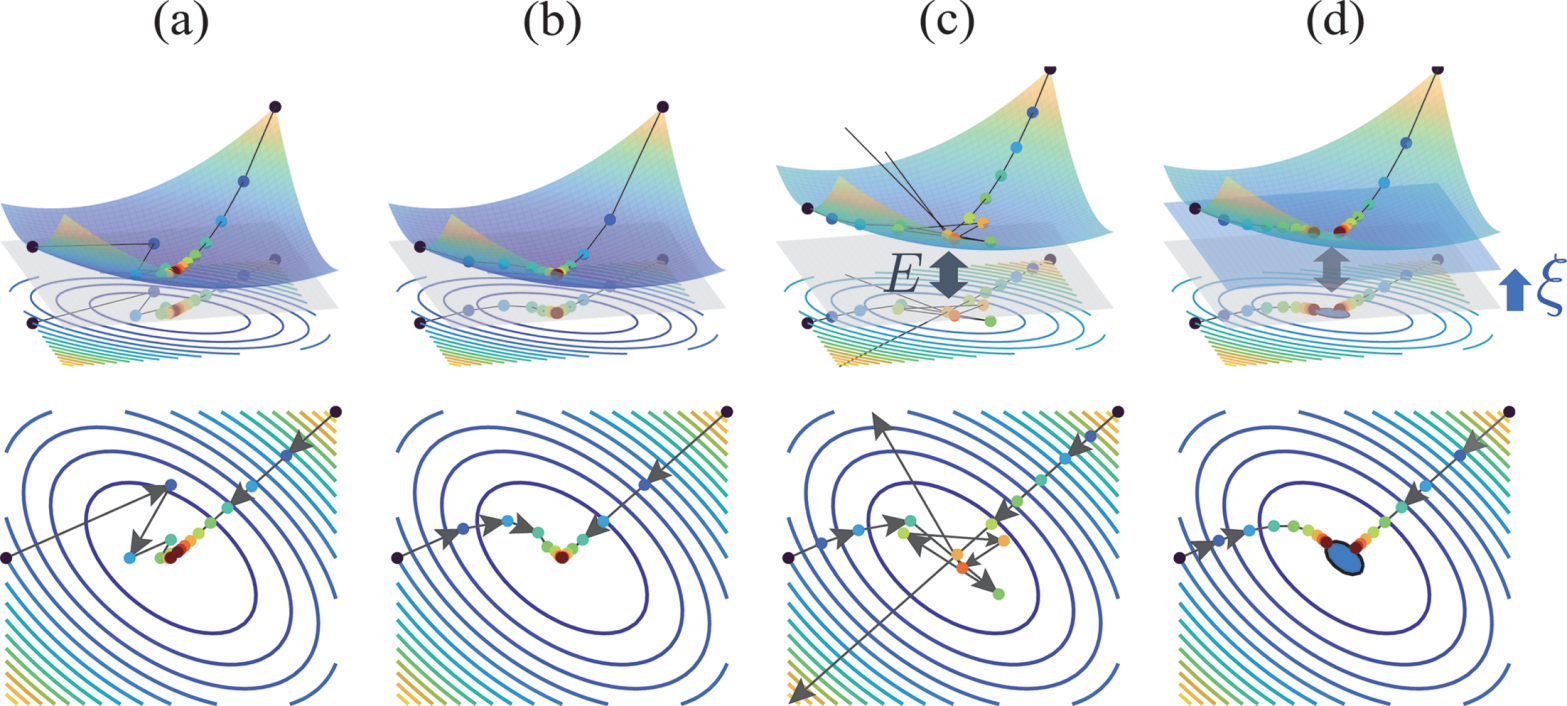
Comparison of iterative updates between the gradient descent method and the subgradient projection for several cases. (*a*) An example using the gradient descent method. (*b*)–(*d*) Examples using the iterative subgradient projection. (*b*) shows the case where the minimum value of the objective function *g* is 0, and (*c*) and (*d*) show the case where the minimum value is *E*. In (*c*) and (*d*), the constants used in the subgradient projection are 0 and ξ, respectively. The three-dimensional schematic diagram in the upper panels is projected to a two-dimensional plane and is shown in the lower panels. The color of the points represents the number of iterations, and the arrows indicate the transition of each update. The plane at *g*(**x**) = 0 is shown in light gray and the plane at *g*(**x**) = ξ in light blue. In the bottom diagram of (*d*), the area where *g*(**x**) ≤ ξ − *E* is shown in blue.

Next, we visualize the importance of the setting of the hyperparameter ξ. Fig. 4 ▸ shows the sequences generated by iterative updates under different conditions of ξ and *E*. As shown in Figs. 4 ▸(*a*) and 4 ▸(*b*), when the minimum value of *g* is 0 and ξ = 0, the sequence of the subgradient projection converges to the minimum in the same way as the gradient method. As shown in Fig. 4 ▸(*c*), when the minimum value of *g* is *E* > 0 and ξ = 0, the sequence diverges because the step size 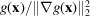 becomes extremely large near the minimum point where the gradient ∇*g*(**x**) is close to 0. To avoid this, we need to set an appropriate constant ξ > 0. As shown in Fig. 4 ▸(*d*), when we set ξ greater than *E*, the points stop at the area where *g*(**x**) ≤ ξ − *E*.

The entire CRISP procedure is summarized in the fol­low­ing pseudocode, where **D**_o_ and **D**_p_ denote the matrices con­tain­ing the updating directions, 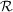 is a set of scan position vectors, and 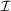 is a set of observed diffraction patterns. The vector **e** stores the cost in each sample for calculation in line 16.
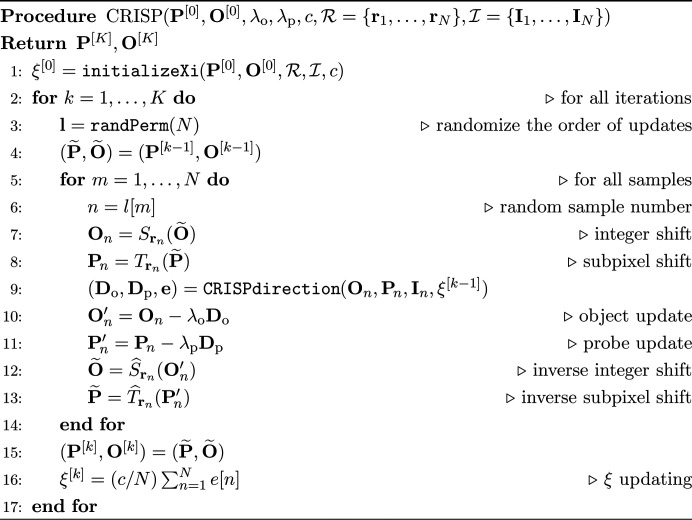
The following subsections are about auxiliary methods for CRISP.

### 3.4. Adaptive step size clipping

As the first auxiliary method, we propose a stabilizing method for updating objects and probes. Although the subgradient projection can avoid local minima, its step size may become extremely large around points where the gradient is close to 0, as shown in Fig. 3 ▸(*b*). This may cause a performance degradation. For stability, limiting the step size can be conceivable. This technique is commonly used in the field of machine learning and has shown good results (Lin *et al.*, 2018 ▸). Fig. 3 ▸(*c*) illustrates updating with subgradient projection with step size clipping. The left sequence in Fig. 3 ▸(*c*) goes to the global minimum while avoiding a large step as in Fig. 3 ▸(*b*). As can be seen, step size clipping may work well for optimization with subgradient projection.

To make it easier to explain step size clipping, we first introduce a step size function *G* that replaces part of the step size in equations (16) and (17) as 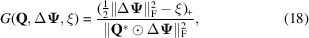
where **Q** is either 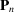 or **O**_*n*_. By using this step size function, the updating formulas of the proposed method in equations (16) and (17) are rewritten as 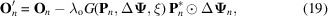

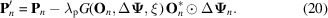
We next introduce a modified step size function that sets an upper limit for the update amount: 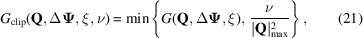
where ν is a clipping parameter that controls the clipping strength. Smaller ν clips the step size more strongly. The clipping parameters for object and probe are denoted by ν_o_, ν_p_, respectively. We use two types of parameter setting in our experiment: (ν_o_, ν_p_) = (1, 1) and (|**P**_*n*_|_max_, |**O**_*n*_|_max_). The former limits the maximum step size to that of ePIE with (α_o_, α_p_) = (λ_o_, λ_p_). This is because 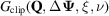 becomes 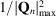, and equation (19) becomes equation (6) when (λ_o_, λ_p_) = (α_o_, α_p_). The latter adopts an adaptive limit that depends on the scale of the object and probe. Our experiment will show that the former setting often works well in practice and that the latter setting may further improve performance. The proposed clipping is used in lines 5 and 6 in the following algorithm, which corresponds to the function CRISPdirection.
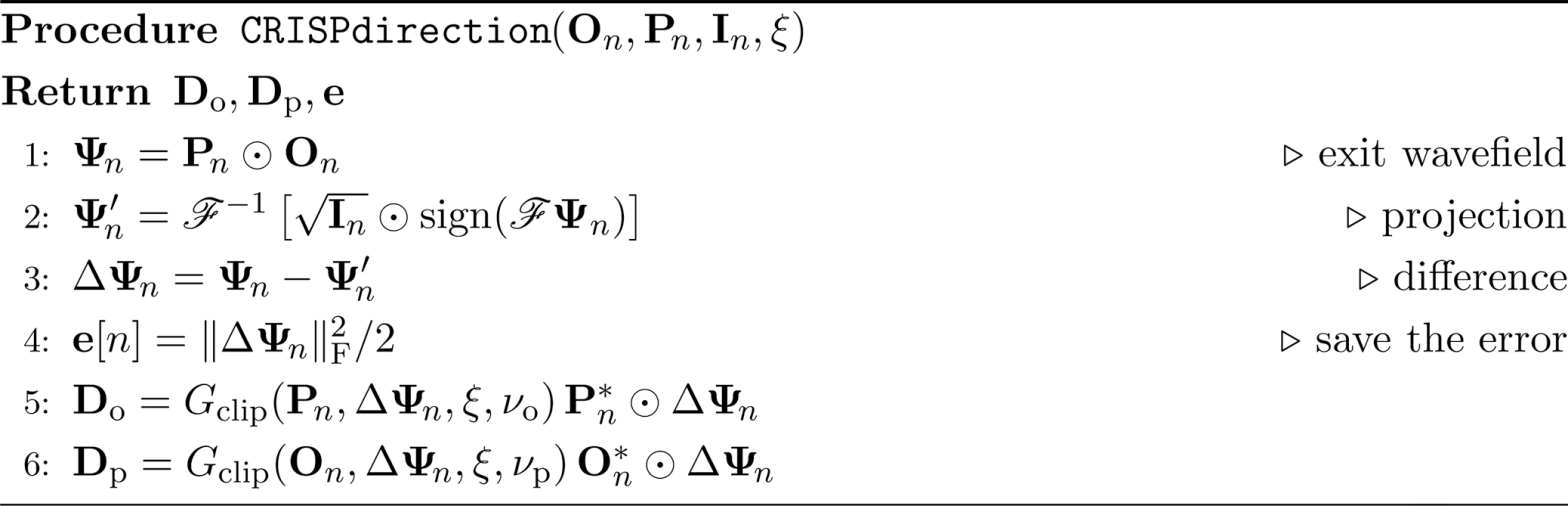


### 3.5. Automatic tuning of ξ

As the second auxiliary method for CRISP, we propose to improve its practicality by relaxing the difficulty of hyperparameter tuning. The subgradient-projection-based update finally stops near a good solution when ξ is appropriately set for the minimum value of the objective function, as shown in Fig. 4 ▸(*d*). However, the update stops in an undesired solution when ξ is set to too large a value because the value of the step size function *G* becomes 0 early before the cost 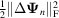 becomes sufficiently small. To avoid this, we automatically adjust ξ with each iteration to keep updating until the cost becomes small.

Since the subgradient-projection-based update depends on the error variable 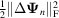, we adjust ξ^[*k*+1]^ according to the average of the cost 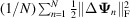 at the *k*th iteration. We adopt the adjusting step as follows: 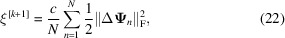
where *c* > 0 is a tuning parameter for adjusting how much to reduce from the average cost. Since we expect the average cost to decrease with updates, we set *c* ∈ (0, 1) so that ξ^[*k*+1]^ becomes smaller than the average cost in the *k*th iteration.

For this procedure, the error should be saved for each iteration as in line 4 in CRISPdirection. In each iteration, ξ is updated to ξ′ after all objects and probes have been updated as line in 16 in the CRISP algorithm. ξ is initialized before the iteration begins as in line 1 in the CRISP algorithm. As shown in the pseudocode initializeXi, this initialization computes the error for each sample using the initial values of the object and probe and then uses all of them to calculate the first ξ.
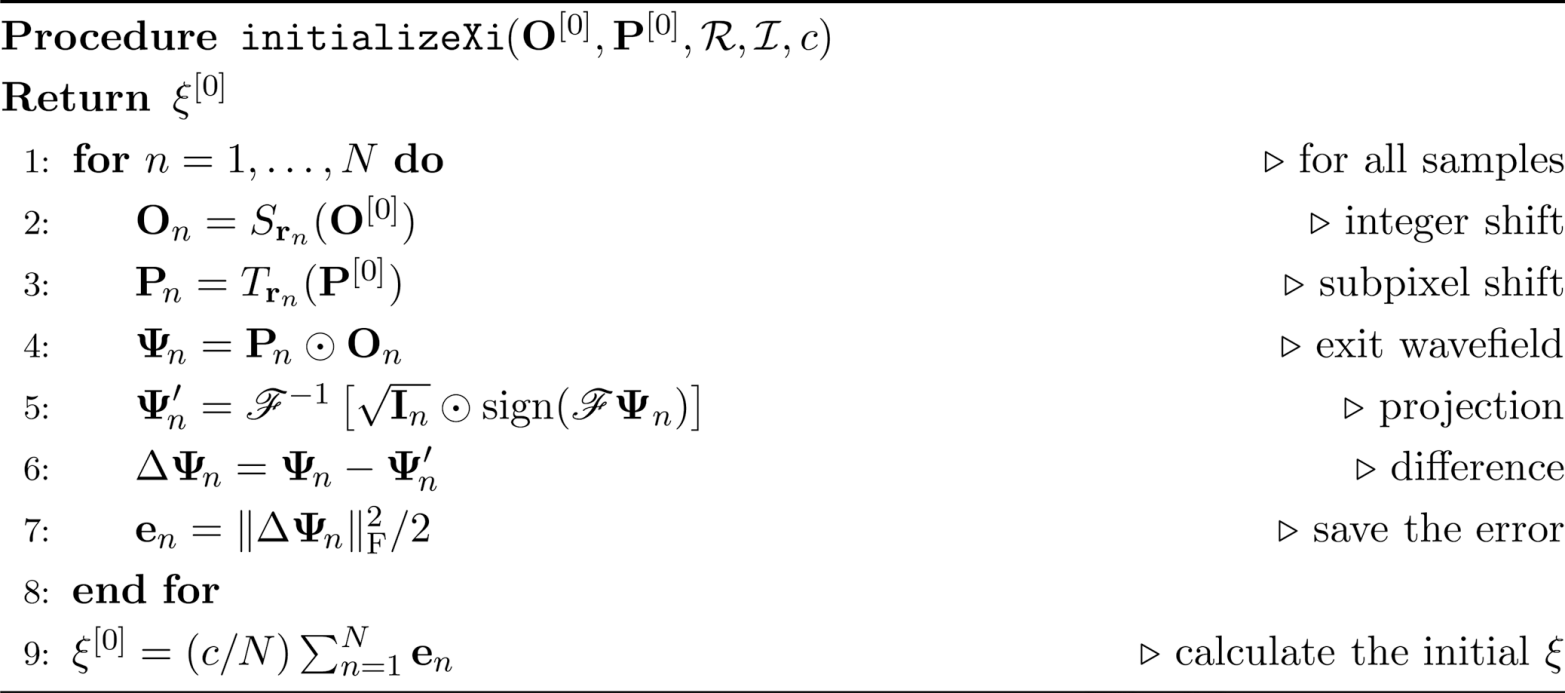


## 4. Experiment

**Figure 5:**
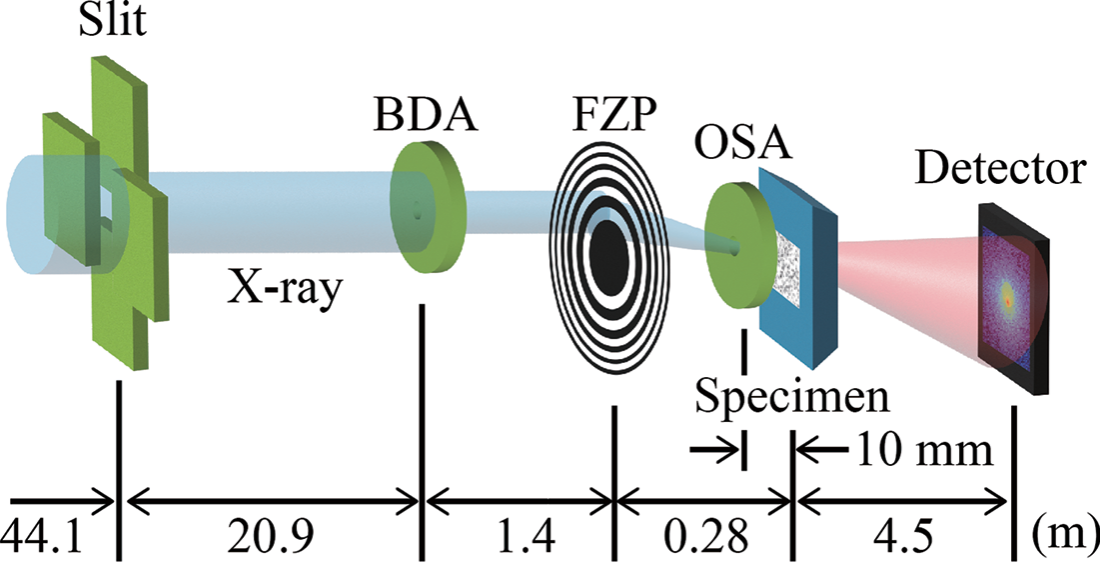
Schematic illustration of the experimental setup.

To investigate the properties of the proposed algorithm, numerical experiments were performed. For a numerical simulation, we used a TiO_2_-particle-filled polymethyl methacrylate film used in a previous paper (Yatabe & Takayama, 2022 ▸). These data were configured to imitate the hard-X-ray ptychographic measurement system at an imaging station of the Hyogo ID beamline BL24XU at SPring-8 (Takayama *et al.*, 2020 ▸, 2021 ▸). This measurement system is shown in Fig. 5 ▸. An undulator radiation X-ray beam monochromatized to a photon energy of 8 keV was cropped with the slit used as a virtual light source, and then the X-ray beam diffracted from the slit illuminated the beam-defining aperture (BDA). The BDA, a Fresnel zone plate (FZP) lens and the specimen were placed according to the thin lens formula; thus the demagnified image of the BDA was formed on the specimen for the local illumination. The first-order diffraction was selected with an order-sorting aperture (OSA). The diameter of the BDA was 20 µm, the outermost zone width and diameter of the FZP were 86 nm and 416 µm, respectively, and the demagnification ratio was set to 5, yielding an illumination beam with a diameter of 4 µm. It was assumed that the diffraction intensity patterns of the specimen were measured with an EIGER X 1M detector (Dectris Ltd) placed 4.5 m downstream of the specimen.

We used two simulation data sets whose diffraction intensities at the origin (*I*_0_) were varied: 10^10^ (high dose) and 10^8^ (low dose). We added Poisson noise to the observed intensity patterns, which is a common assumption for actual measurements. For an experiment with real data, we used the measurement of a Siemens star chart and ink toner particles.

The proposed algorithm CRISP was compared with ePIE. For a quantitative evaluation, we used the following evaluation indices. To check the convergence of the algorithm, the *R*_F_ factor (Miao *et al.*, 2006 ▸) 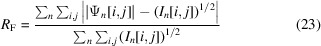
was calculated.

To evaluate the image resolution of the objects, we calculated the Fourier ring correlation (FRC) (Rosenthal & Henderson, 2003 ▸), 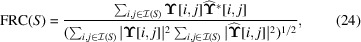
where 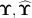 are the Fourier transforms of the estimated and true object transmission functions, respectively, and 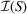 is the set of frequency indices corresponding to the ring whose radius is equal to the given spatial frequency *S*. The closer the value is to 1, the higher the accuracy at that spatial frequency.

### 4.1. Result 1: performance per iteration

We compared the convergence speed among ePIE, rPIE and CRISP. We used the high-dose simulation data. To compare under similar conditions, the step size parameters of the proposed method were set to the same as those of ePIE, *i.e.* α_o_ = λ_o_ = 1.0, α_p_ = λ_p_ = 0.4. The balancing parameters of rPIE were set to those providing the best results according to the preliminary tuning: γ_o_ = 0.1, γ_p_ = 1.0. The clipping parameters and the tuning parameter of CRISP were (ν_o_, ν_p_) = (1, 1) and *c* = 0.5, respectively. The number of iterations was 300. The ratios of the total computational time of CRISP to those of ePIE and rPIE were 1.09 and 1.02, respectively.

**Figure 6:**
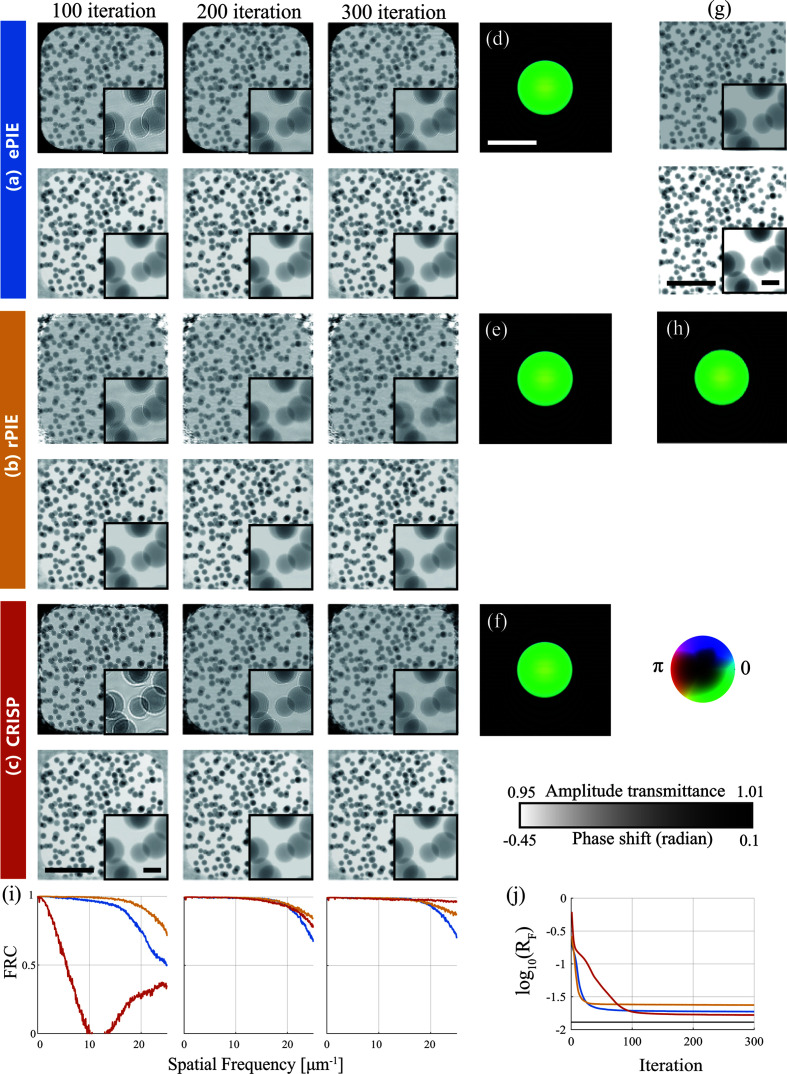
Comparison of performance per iteration among ePIE, rPIE and CRISP. (*a*)–(*c*) show the amplitude (upper) and phase (bottom) of object images reconstructed by (*a*) ePIE, (*b*) rPIE and (*c*) CRISP, respectively. (*d*)–(*f*) show the probe reconstructed by ePIE, rPIE and CRISP, respectively. (*g*) and (*h*) show the ground-truth object image and probe, respectively. (*i*) and (*j*) are the calculated FRC and *R*_F_ factor, respectively. The FRC figures correspond to 100, 200 and 300 iterations from left to right, respectively. The line colors correspond to the colors of (*a*)–(*c*).The horizontal black line in (*j*) represents the *R*_F_ factor between the diffraction patterns and their noise-free version. Bars in (*c*), (*d*) and (*g*) indicate 4 µm, and those in the inset are 0.5 µm.

The results are shown in Fig. 6 ▸. In Fig. 6 ▸(*j*), the *R*_F_ factors of ePIE and rPIE are lower than those of CRISP until the 100th iteration, while CRISP reached a lower *R*_F_ factor than ePIE and rPIE at the 300th iteration. In Fig. 6 ▸(*i*), the FRC of CRISP at the final iteration is higher at the high spatial frequency than that of ePIE and rPIE. The reconstructed-object images shown in Figs. 6 ▸(*a*) to 6 ▸(*c*) reflect the result in Fig. 6 ▸(*i*). At the final (300th) iteration, the amplitude image reconstructed by CRISP was closer to the ground truth [Fig. 6 ▸(*g*)] than that of ePIE. Note that ePIE enhanced the white contour of the particles as shown in the upper-right box of Fig. 6 ▸(*a*), which is known as an artifact of defocusing. Moreover, the amplitude image reconstructed by CRISP shown in the upper-right box of Fig. 6 ▸(*c*) was less noisy than that reconstructed by rPIE shown in the upper-right box of Fig. 6 ▸(*b*). These results confirm that CRISP converges as fast as other methods and can reconstruct higher-quality images.

For each algorithm, the correlation coefficients between the reconstructed probes [Figs. 6 ▸(*d*)–6 ▸(*f*)] and the ground truth [Fig. 6 ▸(*h*)] in the reciprocal space were almost 1, where these were calculated over the spatial frequency range cropped with the aperture of the illumination zone plate.

Compared with ePIE and rPIE, CRISP had a slower drop in *R*_F_ during the first 100 iterations. This behavior can be interpreted as follows. At the beginning of iterations, the error 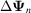 tends to be large, resulting in a large gradient. This makes the step size of ePIE and rPIE large at the beginning of iterations, as shown in Fig. 3 ▸(*a*). On the other hand, the step size of CRISP has less variation for the entire set of iterations, as shown in Fig. 3 ▸(*c*), because equation (15) normalizes its step size by the norm of the gradient in the denominator. These characteristics of CRISP might be the reason for its stable performance and better reconstruction quality.

### 4.2. Result 2: effect of the step size clipping

The following two experiments confirm the validity of the auxiliary methods of CRISP. This experiment verified the effect of the step size clipping. We used both high-dose and low-dose data. To show the difference in behavior due to clipping parameters, we compared the two types of setting: (ν_o_, ν_p_) = (1, 1) (ePIE-like clipping) and (ν_o_, ν_p_) = (|**P**_*n*_|_max_, |**O**_*n*_|_max_) (scale-adaptive clipping). The parameter settings for this experiment with the high-dose data were the same as in Section 4.1. For the experiment with the low-dose data, the step size parameters were set as α_o_ = λ_o_ = 0.4, α_p_ = λ_p_ = 0.2, the balancing parameter for rPIE was γ_o_ = 0.1, γ_p_ = 5.0, the tuning parameter for CRISP was *c* = 0.01, and the number of iterations was 400 (compared with the experiment with high-dose data, more iterations were required for sufficient convergence).

**Figure 7:**
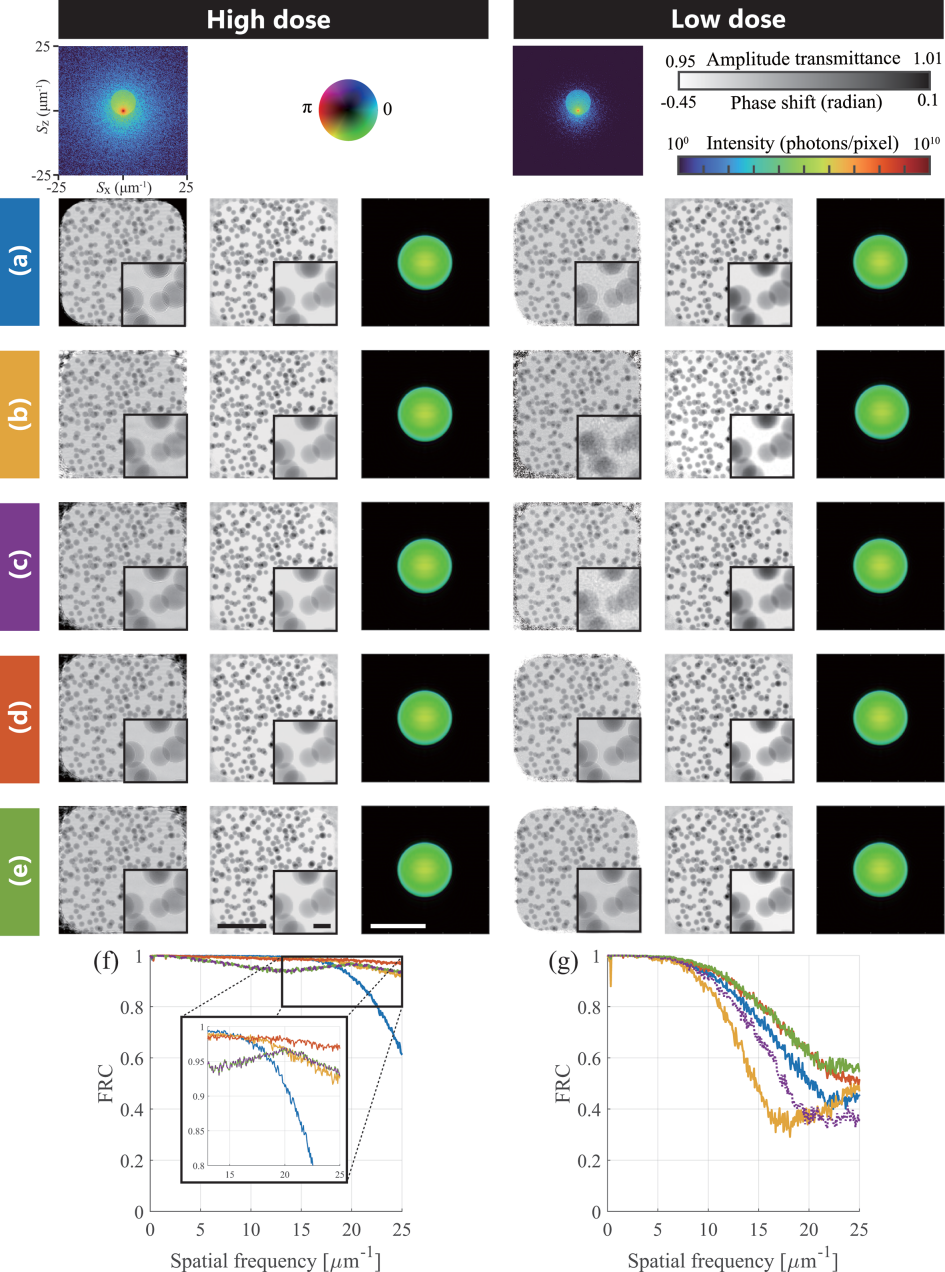
Comparison of reconstruction performance among (*a*) ePIE, (*b*) rPIE and CRISP with (*c*) no clipping, (*d*) ePIE-like clipping and (*e*) scale-adaptive clipping. The left and right columns are high dose and low dose, respectively. The line colors in (*f*) and (*g*) correspond to the colors of (*a*)–(*e*). For ease of viewing, only the purple line is dashed. The reconstructed amplitude and phase images of the object are shown in the left and middle of each column, respectively. The reconstructed probes are shown in the right of each column. (*f*) and (*g*) are the FRC for high dose and low dose, respectively. The typical diffraction intensities are shown at the top. Bars in (*e*) indicate 4 µm, and that in the inset 0.5 µm.

The results are shown in Fig. 7 ▸. In both high-dose and low-dose data, the FRC results with step size clipping were better than those without clipping, and even ePIE and rPIE as shown in Figs. 7 ▸(*f*) and 7 ▸(*g*). The reconstructed-object images, especially those reconstructed from low-dose data, exhibited higher improvement of FRC than those reconstructed without clipping. The third from the right images of Figs. 7 ▸(*d*) and 7 ▸(*e*) were reconstructed with step size clipping. These were less noisy than the image reconstructed without step size clipping [the third from the right of Fig. 7 ▸(*c*)]. These results confirm that step size clipping is essential for CRISP to perform stable high-precision reconstruction. Similarly to Section 4.1, the correlation coefficients in the reciprocal space between the reconstructed probes and the ground truth were 1 for all results.

The behavior of CRISP depended slightly on the clipping parameter as shown in Figs. 7 ▸(*f*) and 7 ▸(*g*). The ePIE-like clipping worked best with the high-dose data, and the scale-adaptive clipping worked slightly better than the ePIE-like clipping with the low-dose data. Although this suggests that which clipping parameter to set may depend on the condition of the data, we found experimentally that ePIE-like clipping generally stabilizes performance. Therefore, CRISP can avoid a detailed setting of the clipping parameter, and the only additional parameter that needs to be set in CRISP is the tuning parameter *c* for automatic tuning.

An advantage of CRISP over rPIE is noise robustness. In Figs. 7 ▸(*f*) and 7 ▸(*g*), rPIE performed well for high-dose data, while FRC was poor for low-dose data. rPIE uses an element-wise step size in equations (10) and (11) to improve the convergence speed, whereas ePIE and CRISP use a common step size for all elements as in equations (6), (7), (19) and (20). Using the common step size may improve the stability of image reconstruction for low-dose data because it balances updates among all elements.

### 4.3. Result 3: effect of automatic tuning

We next verified the effectiveness of the automatic tuning. We compared the reconstruction performance of CRISP with automatic tuning and with an arbitrarily set ξ. We used both the high-dose and low-dose data. The parameter settings were the same as in Section 4.2. In order to confirm the validity of ξ obtained through automatic tuning, we tested it with several constant ξ settings. The constant ξ was set from 0 to 0.75 in increments of 0.05 for the experiment with the high-dose data and from 0 to 0.11 in increments of 0.01 for the experiment with the low-dose data.

**Figure 8:**
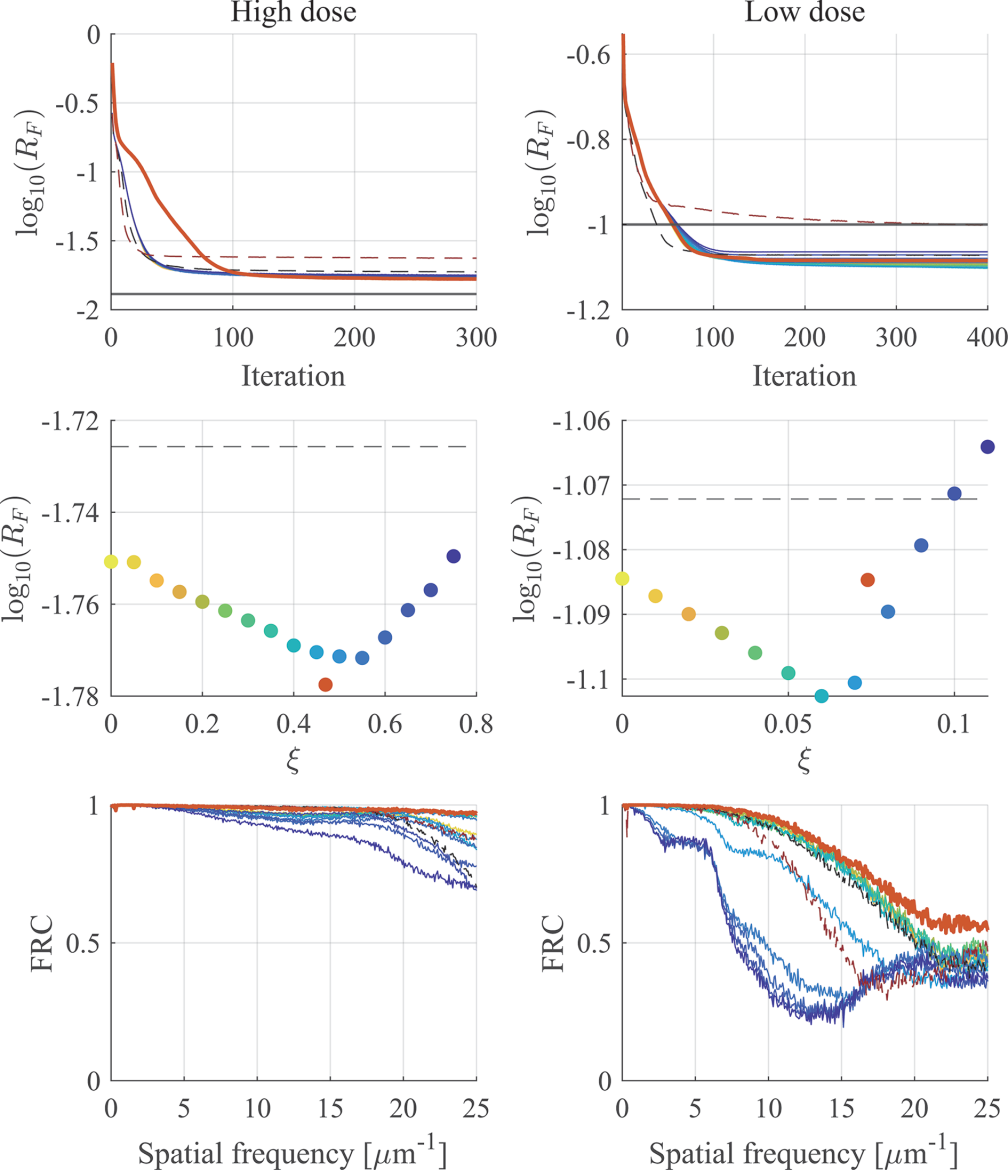
ξ-dependent performance of CRISP. The left and right columns are the result of high-dose and low-dose data, respectively. The top row is the *R*_F_ factor, the middle is the *R*_F_ factor at the final iteration for each ξ and the bottom is FRC. For all subfigures, the colors correspond to values of fixed ξ, which go from yellow to blue as the value increases from 0. The dashed black lines are ePIE, the dashed brown lines are rPIE, the red line and points are the automatic-ξ-tuned CRISP, and the rest are the ξ-fixed CRISP. The black lines in the top row represent the *R*_F_ factor between the diffraction patterns and their noise-free version. The ξ values of the red points on the middle row were calculated using the final updated ξ^[*K*]^ as (1/*c*)ξ^[*K*]^ by considering the scaling by tuning parameter *c*.

The bottom row of Fig. 8 ▸ shows that CRISP with the automatic tuning had the highest FRC at high spatial frequency compared with CRISP with fixed ξ and ePIE for both simulation data sets, while CRISP with better constant ξ also showed higher FRC than ePIE. These results indicate that the automatic tuning allows us to achieve high performance.

We provide further arguments to support the validity of the automatic tuning. The bottom and middle rows of Fig. 8 ▸ show that FRC increased and the *R*_F_ factor decreased as ξ increased to a certain value such as ξ = 0.5 for the high-dose data and ξ = 0.06 for the low-dose data. On the other hand, FRC degenerated when ξ was set greater than its appropriate value. These results suggest that there is a proper ξ range for better performance. The ξ derived by automatic tuning was 0.47 for the high-dose data and 0.074 for the low-dose data, as shown in the middle rows of Fig. 8 ▸. These values were close to the setting of ξ that exhibited the best performance among the fixed ξ. Thus, it was shown that automatic tuning simplifies the difficult parameter setting and can set an appropriate ξ to give a good performance.

### 4.4. Result 4: performance with real data

For real data, we verified the robustness of the proposed method to the order for the object update, which is known to affect the reconstruction performance. We evaluated the dispersion of reconstruction performance in ten trials in which the update order was randomly changed. To evaluate the dispersion, we used the τ_*ij*_ score which is calculated as follows (Sekiguchi *et al.*, 2017 ▸; Takayama & Nakasako, 2024 ▸): 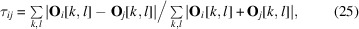
where **O**_*i*_ and **O**_*j*_ are the pair of reconstructed-object images from two different trials. The lower τ_*ij*_ is, the smaller the difference between the two reconstructed images. Since ten trials were conducted, there were 45 combinations of reconstructed images (excluding itself). The step size parameters were set to α_o_ = λ_o_ = 0.8, α_p_ = λ_p_ = 0.4 for the Siemens star chart and α_o_ = λ_o_ = 0.4, α_p_ = λ_p_ = 0.2 for ink toner. The balancing parameters of rPIE were set to γ_o_ = 0.4, γ_p_ = 2.0 for the Siemens star chart and γ_o_ = 0.5, γ_p_ = 1.0 for ink toner. The tuning parameter of CRISP was *c* = 0.01 for both, and the clipping parameter was (ν_o_, ν_p_) = (1, 1) for both. The number of iterations was 300 for the Siemens star chart and 100 for ink toner.

**Figure 9:**
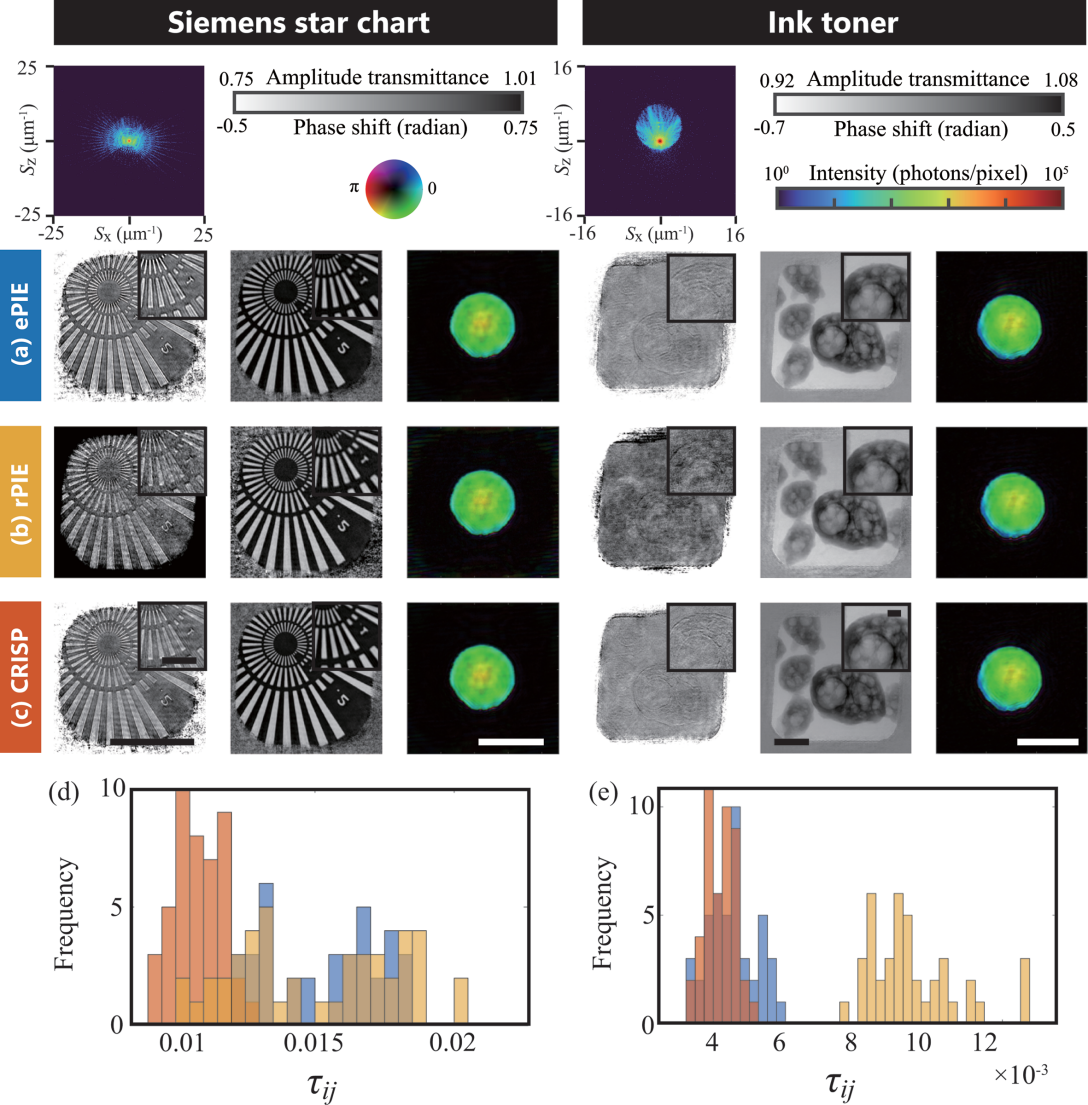
Results for real data. The left and right columns are the Siemens star chart and ink toner, respectively. Typical diffraction intensity patterns are shown at the top. The amplitude images of the object, phase images of the object and probe reconstructed by (*a*) ePIE, (*b*) rPIE and (*c*) CRISP are shown in the left, middle and right, respectively. (*d*) and (*e*) are histograms of τ_*ij*_ for (*d*) the Siemens star chart and (*e*) ink toner. The blue bins are ePIE, the yellow bins are rPIE and the red bins are CRISP. For the Siemens star chart, the median of τ_*ij*_ was 1.4 × 10^−2^ for ePIE, 1.5 × 10^−2^ for rPIE and 1.1 × 10^−2^for CRISP; the variance of τ_*ij*_ was 5.8 × 10^−6^ for ePIE, 8.5 × 10^−6^ for rPIE and 8.0 × 10^−7^ for CRISP; the *R*_F_ factor at the final iteration was 0.422 for ePIE, 0.499 for rPIE and 0.423 for CRISP. For ink toner, the median of τ_*ij*_ was 4.5 × 10^−3^ for ePIE, 9.3 × 10^−3^ for rPIE and 4.2 × 10^−3^ for CRISP; the variance of τ_*ij*_ was 4.2 × 10^−7^ for ePIE, 2.6 × 10^−6^ for rPIE and 1.7 × 10^−7^ for CRISP; the *R*_F_ factor at the final iteration was 0.237 for ePIE, 0.299 for rPIE and 0.230 for CRISP. Bars in (*c*) indicate 4 µm, and that in the inset 1 µm.

The results are shown in Fig. 9 ▸. We visualized the histograms of τ_*ij*_ for each data set as shown in Figs. 9 ▸(*d*) and 9 ▸(*e*). In both figures, τ_*ij*_ scores of CRISP have smaller values and are distributed in a narrower range than those of the other methods. This was also shown by the fact that the median and variance of τ_*ij*_ scores were lower in CRISP than in the other methods.

These results confirm that CRISP is more robust to the update order than the other methods.

## 5. Conclusion

In this paper, we proposed a PR algorithm named CRISP that is based on subgradient projection. It performs error-adaptive updates and can avoid poor update stagnation. The proposed method stably reconstructed higher-quality images than ePIE and rPIE, while the convergence speed and complexity in parameter tuning were almost the same as those of ePIE. In future work, we will extend the proposed method to handle regularizations and different noise models, such as the Poisson model, and apply it to multi-slice approaches.
